# Predictive model for acute respiratory distress syndrome events in ICU patients in China using machine learning algorithms: a secondary analysis of a cohort study

**DOI:** 10.1186/s12967-019-2075-0

**Published:** 2019-10-01

**Authors:** Xian-Fei Ding, Jin-Bo Li, Huo-Yan Liang, Zong-Yu Wang, Ting-Ting Jiao, Zhuang Liu, Liang Yi, Wei-Shuai Bian, Shu-Peng Wang, Xi Zhu, Tong-Wen Sun

**Affiliations:** 1 Department of General ICU, The First Affiliated Hospital of Zhengzhou University, Henan Key Laboratory of Critical Care Medicine, 1 Jianshe East Road, Zhengzhou, 450052 China; 2 Department of Critical Care Medicine, Peking University Third Hospital, Beijing, China; 3 Department of Electrical & Computer Engineering, University of Alberta, Edmonton, Canada; 4 Intensive Care Unit, Beijing Friendship Hospital Affiliated with Capital Medical University, Beijing, China; 5 Intensive Care Unit, Xiyuan Hospital Affiliated with the China Academy of Chinese Medical Sciences, Beijing, China; 6 Intensive Care Unit, Beijing Shijitan Hospital Affiliated with Capital Medical University, Beijing, China; 7 Intensive Care Unit, China-Japan Friendship Hospital, Beijing, China

**Keywords:** Acute respiratory distress syndrome, Machine learning, Predictive model

## Abstract

**Background:**

To develop a machine learning model for predicting acute respiratory distress syndrome (ARDS) events through commonly available parameters, including baseline characteristics and clinical and laboratory parameters.

**Methods:**

A secondary analysis of a multi-centre prospective observational cohort study from five hospitals in Beijing, China, was conducted from January 1, 2011, to August 31, 2014. A total of 296 patients at risk for developing ARDS admitted to medical intensive care units (ICUs) were included. We applied a random forest approach to identify the best set of predictors out of 42 variables measured on day 1 of admission.

**Results:**

All patients were randomly divided into training (80%) and testing (20%) sets. Additionally, these patients were followed daily and assessed according to the Berlin definition. The model obtained an average area under the receiver operating characteristic (ROC) curve (AUC) of 0.82 and yielded a predictive accuracy of 83%. For the first time, four new biomarkers were included in the model: decreased minimum haematocrit, glucose, and sodium and increased minimum white blood cell (WBC) count.

**Conclusions:**

This newly established machine learning-based model shows good predictive ability in Chinese patients with ARDS. External validation studies are necessary to confirm the generalisability of our approach across populations and treatment practices.

## Background

Acute respiratory distress syndrome (ARDS) is a clinical syndrome characterised by tachypnoea, severe hypoxemia, decreased respiratory compliance, and lung tissue damage evident on chest radiographs [1]. Although diffuse alveolar damage is the core pathological process [2], the diagnoses of ARDS and its milder form, acute lung injury (ALI), are based on clinical characterisation. The clinical standards for ALI/ARDS were revised in 2012 and are known as the “Berlin definition” [3]. ARDS is responsible for more than 2 million critical care days and 75,000 deaths in the USA yearly [4] and is associated with an overall mortality ranging from 35 to 50% [5]. Specific and sensitive means of diagnosing ALI/ARDS early are missing, and once diagnosed, it tends to progress quickly. A large number of basic and clinical studies have demonstrated that early diagnosis and intervention are key to improving the survival rate of patients with ARDS [6]. Although it is equally important to predict ARDS events, so far, there have been no reports of models for predicting such cases. Therefore, there is a pressing need for the development and clinical testing of a predictive model for ARDS events, which might improve the clinical diagnosis of ARDS.

According to the 2001 National Institutes of Health definition, a biomarker is “a characteristic that is objectively measured and evaluated as an indicator of normal biological processes, pathogenic processes, or pharmacologic responses to a therapeutic intervention” [7]. Biomarkers reflect pathophysiological mechanisms and, as such, may help in the recognition of ARDS. Combining existing clinical definitions with reliable biomarkers may therefore enhance the diagnosis of ARDS. In addition to the recognition of ARDS, biomarkers may contribute to risk stratification and the prediction of outcomes or serve as surrogate endpoints to monitor interventions [8]. The proposed advantages of biomarkers [8], together with the limited reliability and validity of the American–European Consensus Criteria (AECC) criteria [9, 10], have spurred the search for reliable ARDS biomarkers during the last two decades. Many biomarkers for the diagnosis of ARDS have been found, such as the receptor for advanced glycation end-products (RAGE), angiopoietin-2 (Ang-2), surfactant protein D (SP-D) and inflammatory factors [interleukin (IL)-6, IL-8, and tumour necrosis factor-α (TNF-α)] [11, 12]. However, no sensitive and specific clinical biomarkers for ARDS have been found [13].

In this secondary analysis of a prospective and independent cohort study, the primary goal was to find several new biomarkers that differ from the previously studied biomarkers for ARDS and to establish a reliable predictive model for ARDS events that includes these new biomarkers.

## Methods

### Study population and ARDS definition

This study was a secondary analysis of a prospective observational study [14] conducted from January 1, 2011, to August 31, 2014, in five intensive care units (ICUs) in the Beijing metropolitan area: Peking University Third Hospital northwest of Beijing, Beijing Friendship Hospital to the south, Beijing Shijitan Hospital in the center, Beijing Xiyuan Hospital to the west, and China–Japan Friendship Hospital in the northeast (Clinicaltrials.gov Identifier: NCT02944279).

Each ICU admission was screened for eligible participants. The exclusion criteria were age < 18 years; history of chronic lung diseases, such as pulmonary fibrosis or bronchiolitis; history of pneumonectomy; treatment with immunomodulating therapy other than corticosteroids, such as granulocyte colony stimulating factor, cyclophosphamide, cyclosporine, interferon, or TNFα antagonists; presence of other immunodeficient conditions, such as HIV infection, leukaemia, or neutropenia (absolute neutrophil count < 1000/mL); history of organ or bone marrow transplants other than an autologous bone marrow transplant; directive to withhold intubation; ICU stay duration < 72 h; or development of ARDS before ICU admission. Patients at risk for developing ARDS were defined as critically ill patients with at least one of the following conditions predisposing them to developing ARDS: sepsis; septic shock; trauma; pneumonia; aspiration (indicated inhalation of gastric juice, fresh water, seawater, amniotic fluid, etc.); massive transfusion of packed red blood cells (PRBCs; defined as > 8 PRBC units in the 24-h period prior to admission); or severe pancreatitis. After selection, patients at risk for developing ARDS were followed daily and assessed according to the Berlin definition [3]. All patients were followed until hospital discharge or death within 60 days from the first day of study enrolment. The full methodological details of this cohort study have been previously published [14]. In this secondary analysis, we used only the variables from the first day of admission before the patient developed ARDS to build this prediction model. In addition, for several variables, such as heart rate, respiratory rate, temperature, glucose, haematocrit, and sodium, we used only the minimum or maximum value from multiple measurements. The ensemble model was written in the Python scripting language (version 3.6.5, Python Software Foundation, Wilmington, DE, USA, https://www.python.org).

### Statistical analysis

The binary variables are described as counts and percentages and were evaluated by the Chi-squared test or Fisher’s exact test. Continuous variables of each group are presented as the mean ± SEM. Student’s t-test was used to compare the normally distributed continuous variables; otherwise, the Mann–Whitney U test was used. *P *< 0.05 was considered statistically significant. All analyses were performed using SPSS 21.0 (SPSS, Chicago, IL).

### Predictive model development

In this study, we aimed to construct an ensemble model called a random forest model that consisted of a population of decision-tree classifiers. In the forest, each decision-tree classifier was built with a bootstrap sample of features and independent observations. As a result, random forests can avoid overfitting and yield an overall improved model with a high predictive accuracy because the randomness makes the model less sensitive to variation [15]. Notably, the implementation of the combination used in this study replaces voting on each decision-tree classifier by averaging their probabilistic prediction to decrease the variance [16–18]. In general, there are two key parameters used in the design of random forests: (i) the number of decision trees and (ii) the size of the random subsets of features. In most cases, more trees in the forest produce more robust predictive accuracy but require a longer computation time. The latter controls the trade-off between variance and bias. From empirical and clinical research, the number of decision trees and the size of the random subset are set to 100 and the square root of the number of features, respectively. The whole process of constructing a random forest algorithm can be described briefly by the following steps: (i) select “*k*” features from the training set as a subset; (ii) calculate the node by using the best split among the “*k*” features; (iii) create child nodes by using the best split; (iv) repeat from step (i) to step (iii) until the iteration ending conditions (the iteration of the above process repeated 1000 times) are met; and (iv) repeat from step (i) to step (iv) until 100 decision trees are archived. After building the random forest, the predictions are made with testing data by using the average of these individual tree outputs. The ensemble model was written in the Python scripting language (version 3.6.5, Python Software Foundation, Wilmington, DE, USA, https://www.python.org). The 296 selected patients were randomly divided into training (ARDS = 76 and non-ARDS = 160) and testing (ARDS = 15 and non-ARDS = 45) sets at a ratio of 4:1. The training set was used to build the ensemble model, while the testing set was used to evaluate the predictive performance of the model. In this study, the ensemble random forest algorithm was also used to predict the accuracy of the models based on different subsets of features. Because the relative rank of each feature could be used to reflect the relative importance of features to the ratings of overall prediction performance [16–18], we applied a random forest algorithm to rank the contribution of each feature, constructed models on the feature subspaces and provided a comparison of the corresponding model quality scores using testing data. In addition to the classification accuracy and the area under the receiver operating characteristic (ROC) curve (AUC), the Matthews correlation coefficient (MCC) and F-measure ($F_{1}$) were also used to evaluate the performance of the constructed model.

$$ MCC = \frac{TP \times TN - FP \times FN}{{\sqrt {\left( {TP + FP} \right)\left( {TP + FN} \right)\left( {TN + FP} \right)\left( {TN + FN} \right)} }} $$

$$ F_{1} = 2 \cdot \frac{precision \cdot recall}{precision + recall} $$

$$ precision = \frac{TP}{TP + FP}\quad {\text{and }}\quad  recall = \frac{TP}{TP + FN} $$

Here, $TP$, $TP$, $TN$ and $FN$ indicate the number of correctly identified ARDS patients (true positive; $TP$), the number of non-ARDS patients who were identified as having ARDS (false positive; $FP$), the number of non-ARDS patients who were identified as having non-ARDS (true negative; $TN$) and the number of ARDS patients who were identified as having non-ARDS (false negative; $FN$).

### Patient and public involvement

In this study, we used deidentified data from the original cohort study with no direct involvement of or interaction with participants in the design, recruitment or conduct of this study.

## Results

### Patient characteristics

**Fig. 1:**
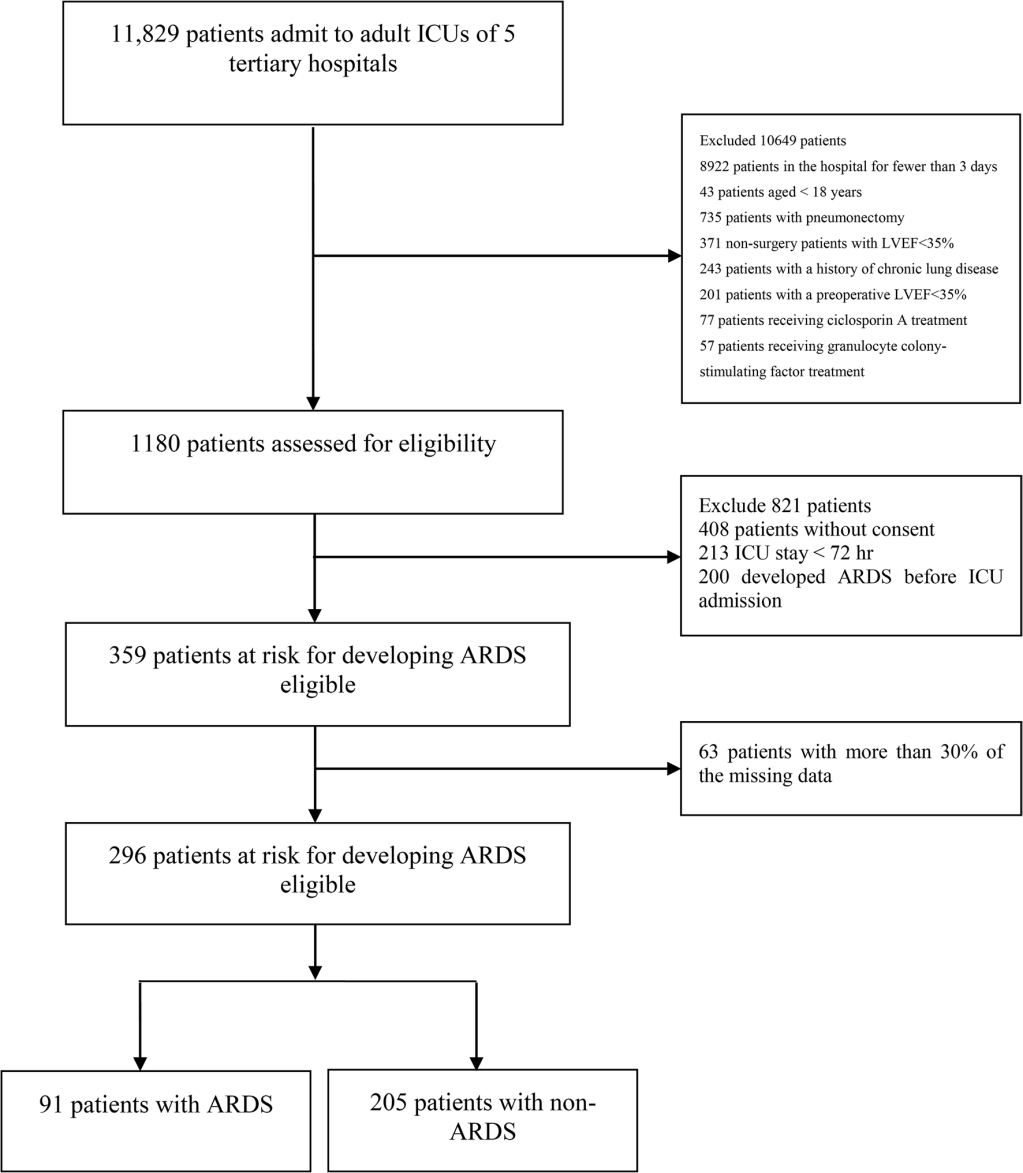
Flow chart of the study selection

A total of 11,829 patients were admitted to the ICU, and 296 patients (203 men, 93 women; mean age, 65.40 ± 18.13 years) were included in this study. Among them, 91 (30.74%) developed ARDS. Table 1 shows the baseline characteristics and clinical/laboratory parameters in the training set. A total of 42 variables, including baseline characteristics, clinical/laboratory parameters, and predisposing conditions, were collected for each patient; many other variables with several missing values were omitted. The basic information compared between the training and validation sets is shown in Table 2. Figure 1 shows the process of cohort selection.

**Table 1:** Baseline characteristics and clinical and laboratory parameters in the training dataset

Variable	Non-ARDS (n = 160)	ARDS (n = 76)	*P* value
Age (year)	63.89 ± 18.00	68.07 ± 17.86	0.096
Sex (male)	108 (67.5%)	59 (77.6%)	0.127
Bacteraemia	2 (1.25%)	2 (2.63%)	0.596
Sepsis	127 (79.4%)	64 (84.2%)	0.479
Septic shock	55 (34.4%)	30 (39.5%)	0.470
Pneumonia	84 (52.5%)	43 (56.6%)	0.579
Vasopressor^a^	69 (43.1%)	35 (46.1%)	0.677
Fracture	3 (1.88%)	1 (1.32%)	1.000
Pulmonary contusion	0 (0%)	3 (3.95%)	0.033*
Aspiration	5 (3.13%)	5 (6.58%)	0.299
Multiple transfusion	15 (9.38%)	9 (11.84%)	0.646
Previous ARDS	0 (0%)	2 (2.63%)	0.103
Autoimmune disease	0 (0%)	0 (0%)	–
Diabetes	30 (18.75%)	12 (15.79%)	0.716
Previous sepsis	0 (0%)	1 (1.32%)	0.322
Tobacco	53 (33.13%)	32 (42.11%)	0.194
Familial diabetes mellitus	13 (8.13%)	3 (3.95%)	0.280
Leukaemia	0 (0%)	1 (1.32%)	0.322
Dialysis	3 (1.88%)	2 (2.63%)	0.658
Metastatic solid tumour	8 (5%)	2 (2.63%)	0.507
Immunosuppression	1 (0.63%)	0 (0%)	1.000
Hepatic encephalopathy	1 (0.63%)	0 (0%)	1.000
Hepatocirrhosis	2 (1.25%)	0 (0%)	1.000
Alcohol abuse for 12 months	18 (11.25%)	7 (9.21%)	0.821
APACHE II score	19.98 ± 5.77	19.92 ± 5.94	0.947
PH	7.38 ± 0.09	7.37 ± 0.11	0.509
Minimum systolic pressure	102.45 ± 20.62	92.97 ± 18.70	0.001*
Maximum systolic pressure	140.76 ± 23.17	138.20 ± 26.63	0.450
Minimum MAP	78.25 ± 72.22	69.20 ± 14.88	0.281
Maximum MAP	98.21 ± 16.92	97.53 ± 17.76	0.777
Minimum heart rate	91.41 ± 16.80	87.04 ± 18.85	0.074
Maximum heart rate	122.19 ± 20.30	122.62 ± 23.18	0.886
Minimum respiratory rate	22.22 ± 4.65	25.08 ± 4.40	0.000*
Maximum respiratory rate	31.08 ± 5.70	34.50 ± 6.23	0.000*
Minimum temperature	36.73 ± 0.92	36.63 ± 0.79	0.412
Minimum creatinine	1.44 ± 1.43	1.36 ± 0.98	0.655
Maximum creatinine	1.56 ± 1.49	1.50 ± 1.07	0.754
Minimum glucose	140.32 ± 104.32	123.79 ± 58.33	0.199
Minimum haematocrit	30.13 ± 6.37	29.13 ± 7.10	0.282
Minimum white blood cell count	11.55 ± 5.72	12.63 ± 7.20	0.253
Minimum sodium	140.67 ± 6.34	138.80 ± 6.25	0.034*
Minimum potassium	3.97 ± 0.68	3.89 ± 0.55	0.366

The binary variables are described as counts and percentages and were evaluated by the Chi-squared test or Fisher’s exact test. Continuous variables of each group are presented as the mean ± SEM. Student’s t-test was used to compare the normally distributed continuous variables

*ARDS* acute respiratory distress syndrome, *MAP* mean arterial pressure, *APACHE II* Acute Physiology and Chronic Health Evaluation II

*P < 0.05, ARDS compared with non-ARDS

^a^Vasopressor use before and 24 h after entering the ICU

**Table 2:** Baseline characteristics and clinical/laboratory parameters in the training and testing cohorts

Variable	Training cohorts (n = 236)	Testing cohorts (n = 60)	*P* value
Sex (male)	167 (70.8%)	36 (60%)	0.121
Age (year)	65.23 ± 18.02	66.03 ± 18.68	0.761
Minimum respiratory rate	23.14 ± 4.75	23.62 ± 5.08	0.494
Maximum respiratory rate	32.18 ± 6.08	31.65 ± 6.25	0.551
Minimum haematocrit	29.81 ± 6.62	30.52 ± 6.72	0.461
Minimum systolic pressure	99.40 ± 20.47	91.82 ± 19.50	0.010*
Minimum MAP	75.33 ± 60.14	67.25 ± 15.10	0.303
Maximum heart rate	122.33 ± 21.22	126.18 ± 21.37	0.211
Minimum glucose	135.00 ± 92.24	124.03 ± 54.68	0.378
Minimum white blood cell count	11.90 ± 6.24	12.88 ± 7.29	0.295
Minimum heart rate	90.00 ± 17.57	93.88 ± 17.85	0.129
Minimum temperature	36.70 ± 0.88	36.66 ± 0.84	0.738
Minimum sodium	140.06 ± 6.35	139.87 ± 6.82	0.834
APACHE II	19.96 ± 5.81	20.78 ± 5.51	0.322
PH	7.38 ± 0.10	7.37 ± 0.10	0.283
Bacteraemia	4 (1.69%)	3 (5%)	0.150
Diabetes	42 (17.8%)	17 (28.3%)	0.073
Tobacco	85 (36.0%)	24 (40%)	0.653

The binary variables are described as counts and percentages and were evaluated by the Chi-squared test or Fisher’s exact test. Continuous variables of each group are presented as the mean ± SEM. Student’s t-test was used to compare the normally distributed continuous variables. *P < 0.05, ARDS compared with non-ARDS

*MAP* mean arterial pressure, *APACHE II* Acute Physiology and Chronic Health Evaluation II

### Key features and classification results

**Fig. 2:**
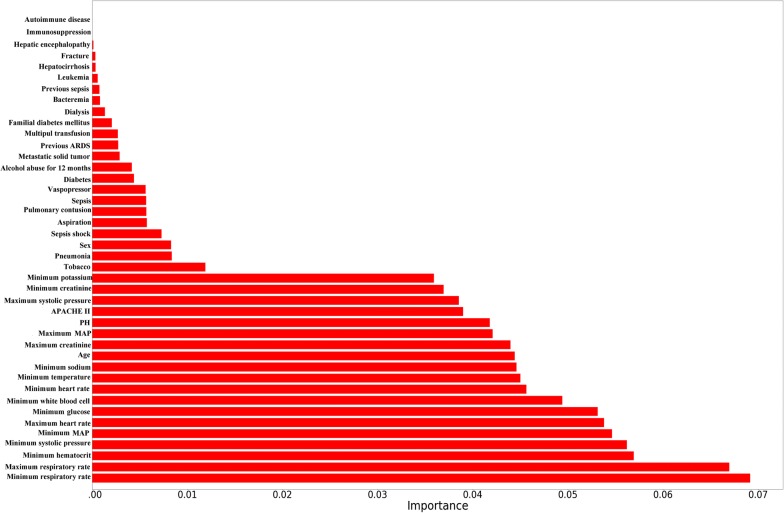
Importance of the 11 variables included in the predictive model for ARDS events. *ARDS* acute respiratory distress syndrome, *MAP* mean arterial pressure, *APACHE II* Acute Physiology and Chronic Health Evaluation II

In most cases, an ensemble model with a greater number of variables will provide a more accurate prediction than a model with fewer variables. However, it is more cost-effective and efficient to obtain similar or even the same improvement by using prominent features, which can thus benefit clinical practice. Based on the fact that features built on the top of trees contribute more to predicting ARDS in at-risk patients, the relative importance of each feature is provided in Fig. 2.

**Fig. 3:**
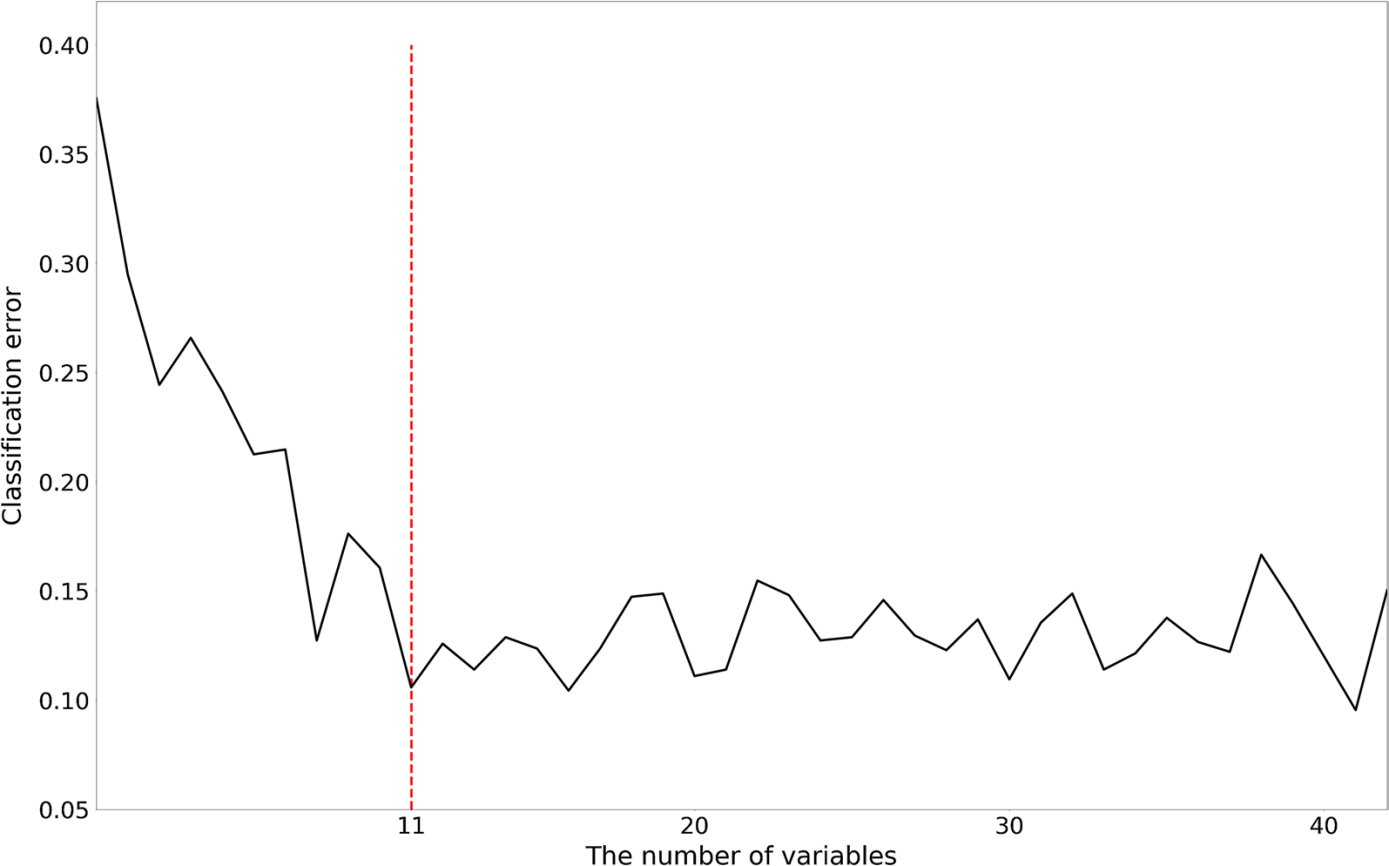
Relationship between the number of variables and classification error

**Fig. 4:**
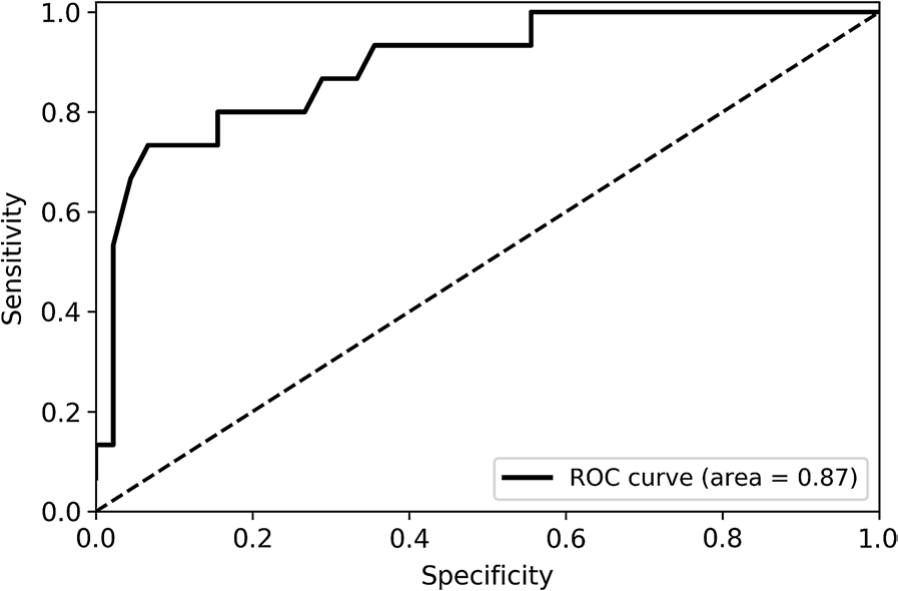
ROC curve (of the testing set) for predicting ARDS events using the predictive model. *ROC* receiver operating characteristic

Next, we performed random forest classification with the same parameters (to make the comparison possible and remove the effect of the parameters) with different subsets of features to calculate the changes in AUC values, as illustrated in Fig. 3. In this study, the AUC values of different feature combinations determined the importance of the input variables. As shown in Fig. 3, the classification error decreases as the number of features gradually increases. The AUC value remains at a similar level after the number of features increases past 11. Therefore, the following 11 features were included in the final model for the prediction of ARDS: minimum respiratory rate, maximum respiratory rate, minimum haematocrit, minimum systolic blood pressure, minimum mean arterial pressure (MAP), maximum heart rate, minimum glucose, minimum white blood cell (WBC) count, minimum heart rate, minimum temperature, and minimum sodium level. With the testing set, the final predictive model achieved an AUC of 0.87 (ROC curve illustrated in Fig. 4), an accuracy of 82%, an MCC of 0.64 and an F1 of 0.73; these results are sufficient to predict which patients will develop ARDS. To demonstrate the robustness of the predictions of the model, the final ensemble model with 11 features included 200 bootstrap replicates [19–21] and achieved an average AUC of 0.82 (with an average accuracy of 0.83, an average MCC of 0.50 and an average F1 of 0.57) in the testing set. The prediction results suggest that the ensemble model with 11 key features is feasible and practical.

## Discussion

This study presents the first predictive model including 11 predictors for ARDS events. Specifically, the 11 predictors included the following: maximum and minimum respiratory rate and heart rate as well as minimum systolic blood pressure, MAP, temperature, WBC count and the levels of glucose, haematocrit, and sodium. Furthermore, the maximum and minimum respiratory rate and the minimum systolic blood pressure on the first day of admission were significantly associated with ARDS events. In addition, for the first time, four new biomarkers were included in the predictive model for ARDS events: decreased minimum haematocrit, glucose, and sodium levels as well as increased minimum WBC count.

Acute respiratory distress syndrome is a life-threatening inflammatory disease of the lungs [22, 23]. Although a mechanical ventilation strategy has been shown to influence mortality in this syndrome, there is currently no proven pharmacologic treatment despite more than 30 completed or ongoing clinical trials [22]. However, many studies [24–28] have reported different predictive models for in-hospital mortality in ARDS patients, and several studies [22, 29–33] have also shown that there are many predictors of mortality in ARDS patients. Terpstra et al. [12] reported 20 biomarkers for the diagnosis of ARDS and 19 biomarkers for predicting mortality in ARDS patients. In addition, some studies [34, 35] have shown that combining multiple biomarkers can enhance diagnostic accuracy. In the present study, we established a predictive model for ARDS events in ICU patients.

In our study, we selected 11 prominent predictors from 42 variables for the predictive model of ARDS events. Previous studies [36–38] have reported that a majority of predictors of mortality or factors involved in diagnosis in ARDS patients are inflammatory factors or lung surface proteins; however, the predictors that we selected are biochemical indicators of ARDS events. Moreover, we included four basic vital signs in the predictive model for ARDS events and found that the minimum and maximum respiratory rates were increased in critical patients with ARDS or non-ARDS compared with healthy patients and were higher in ARDS patients than in non-ARDS patients. In addition, the minimum systolic pressure and MAP were lower in critical patients with ARDS or non-ARDS than in healthy patients and lower in ARDS patients than in non-ARDS patients, which is consistent with the clinical manifestations of ARDS [39]. Furthermore, this is the first model to include four new biomarkers as predictors of ARDS events. First, the minimum glucose level was tested in our model for ARDS patients; glucose levels were higher in critical patients with ARDS or non-ARDS than in healthy people and lower in ARDS patients than non-ARDS patients. Inflammation plays a vital role in ARDS events [40], and many studies [41, 42] have shown a protective effect of hyperglycaemia against ARDS due to inhibition of the protein nuclear factor-kappa-B (NF-κB) inhibitor alpha (IκB-α) and the p56 subunit and the impairment of NF-κB activation in sepsis-induced ALI/ARDS; on the other hand, high glucose levels are associated with decreased neutrophil migration, decreased inflammatory factor secretion, and a reduced inflammatory response. Moreover, a meta-analysis [43] also reported that the risk of death was decreased in adult ARDS patients with pre-existing diabetes, supporting the protective effect of hyperglycaemia against ARDS; this finding was in line with the results of the lung injury prediction score (LIPS) [44, 45]. All of the aforementioned research supports the results of our study. Second, the minimum sodium level was within the normal range but was lower in ARDS patients than in non-ARDS patients. This result may be associated with inhibited lung epithelial sodium channels (ENaCs) in ARDS patients. Several studies [46–50] have reported that inflammation alters the functions of ENaC and ATPase, inhibiting the active transport of Na^+^ from the alveoli to the interstitium, increasing the exchange of sodium in the vasculature and lung interstitium, and ultimately reducing the sodium concentration in the vasculature. In addition, another study [51] showed that pharmacological inhibitors of lung apical Na^+^ channels can reduce the rate at which fluid is cleared and form a positive feedback loop with inflammation in the lung, which may also explain the results of our study. Third, the minimum WBC count was within the normal range but was higher in ARDS patients than non-ARDS patients. WBCs may be regarded as the most important effector cells involved in acute inflammation during the pathogenesis of ARDS. In the case of trauma, sepsis, acute pancreatitis, physical and chemical stimulation, or extracorporeal circulation, as a result of the effects of lipopolysaccharide, complement component 5a receptor, and IL-8, WBCs are concentrated in pulmonary capillaries. Furthermore, WBCs can adhere to endothelial cells and migrate across the endothelium and then enter the lung interstitium, which leads to WBC movement to the alveolar cavity from the alveolar epithelium. Furthermore, there are many types of adhesion molecules involved in this process. Finally, stimulated alveolar macrophages (AMS) release IL-1, TNF-α and IL-8, which promote the chemotaxis and aggregation of WBCs in the lung and may promote ALI; this finding is consistent with the fact that ARDS is associated with an inflammatory environment in the lung [52–54]. The evidence from the above studies is insufficient, although they provide insight into the mechanism underlying ARDS. Most importantly, some recent studies [55, 56] have developed a model of ARDS sub-phenotypes that not only reflects the developmental tendency of ARDS but also plays a decisive role in clinical treatment. Fourth, the minimum haematocrit level was within the normal range but was lower in ARDS patients than in non-ARDS patients. The mechanism underlying this result may be explained by a study [57] showing that the systemic blood flow rate per unit body surface decreases significantly from baseline following the induction of ARDS and that the haematocrit level increased as the systemic blood flow decreased, effectively increasing the systemic oxygen delivery within a certain range in ARDS patients; this process is in accordance with our study results. In sum, we believe that the biomarkers newly discovered in this study provide guidance for future interventional research on ARDS.

In addition, this secondary analysis has several limitations. First, we defined ARDS based only on the Berlin Definition, which varies from the definition of the AECC [3], which may increase the difficulty of diagnosis and the omission of some patients who developed ARDS during the study. Second, this study is a secondary analysis of data from a prospective observational study that was not recorded and indicated when the patients developed ARDS. Third, this prediction model may lack generalisability because the 42 included variables are still too few and because many other variables with too many missing values were omitted. The greater the number of included variables, the higher the predictive accuracy of this model. However, we hope that we can include more patients and variables in future prospective research. Fourth, the robustness of this study cannot be confirmed without an external validation cohort. We hope to accomplish this aim in future prospective research.

## Conclusions

A model with 11 key features was successfully established for predicting ARDS events in Chinese patients. This model can be applied to predict ARDS events by using biomarkers, such as minimum WBC count and glucose, haematocrit and sodium levels. Four new biomarkers were included in this model: decreased minimum sodium concentration, haematocrit, and glucose levels and increased minimum WBC count.

## Abbreviations

ARDS: acute respiratory distress syndrome
ICUs: intensive care units
ROC: receiver operating characteristic
AUC: area under the receiver operating characteristic curve
WBC: white blood cell
ALI: acute lung injury
AECC: American–European Consensus Criteria
RAGE: receptor for advanced glycation end-products
SP-D: surfactant protein D
IL: interleukin
TNF-α: tumour necrosis factor-α
PRBCs: packed red blood cells
MCC: Matthews correlation coefficient
F_1_: F-measure
TP: true positive
FP: false positive
TN: true negative
FN: false negative
MAP: mean arterial pressure
APACHE II: Acute Physiology and Chronic Health Evaluation II
NF-κB: nuclear factor-kappa-B
IκB-α: inhibition of the protein nuclear factor-kappa-B inhibitor alpha
ENaCs: epithelial sodium channels
AMS: alveolar macrophages
